# *In vitro* studies on the sensitivity pattern of *Plasmodium falciparum* to anti-malarial drugs and local herbal extracts

**DOI:** 10.1186/1475-2875-13-63

**Published:** 2014-02-20

**Authors:** Grace I Olasehinde, Olusola Ojurongbe, Adegboyega O Adeyeba, Obasola E Fagade, Neena Valecha, Isaac O Ayanda, Adesola A Ajayi, Louis O Egwari

**Affiliations:** 1 Department of Biological Sciences, Covenant University, Ota, Ogun State, Nigeria; 2 Department of Medical Parasitology, LAUTECH Teaching Hospital, Osogbo, Osun State, Nigeria; 3 Department of Botany, University of Ibadan, Ibadan, Oyo State, Nigeria; 4 National Institute of Malaria Research, New Delhi, India

**Keywords:** *In vitro*, Sensitivity, Anti-malarial, Local herbs, Isolates

## Abstract

**Background:**

The resistance of human malaria parasites to anti-malarial compounds has become considerable concern, particularly in view of the shortage of novel classes of anti-malarial drugs. One way to prevent resistance is by using new compounds that are not based on existing synthetic antimicrobial agents.

**Results:**

Sensitivity of 100 *Plasmodium falciparum* isolates to chloroquine, quinine, amodiaquine, mefloquine, sulphadoxine/pyrimethamine, artemisinin, *Momordica charantia* (‘Ejirin’) *Diospyros monbuttensis* (‘Egun eja’) and *Morinda lucida* (‘Oruwo’) was determined using the *in vitro* microtest (Mark III) technique to determine the IC_50_ of the drugs. All the isolates tested were sensitive to quinine, mefloquine and artesunate. Fifty-one percent of the isolates were resistant to chloroquine, 13% to amodiaquine and 5% to sulphadoxine/pyrimethamine. Highest resistance to chloroquine (68.9%) was recorded among isolates from Yewa zone while highest resistance to amodiaquine (30%) was observed in Ijebu zone. Highest resistance to sulphadoxine/pyrimethamine was recorded in Yewa and Egba zones, respectively. A positive correlation was observed between the responses to artemisinin and mefloquine (P<0.05), artemisinin and quinine (P<0.05) and quinine and mefloquine (P<0.05). A negative correlation was observed between the responses to chloroquine and mefloquine (P>0.05). Highest anti-plasmodial activity was obtained with the ethanolic extract of *D. monbuttensis* (IC_50_ = 3.2nM) while the lowest was obtained from *M. lucida* (IC_50_ =25nM).

**Conclusions:**

Natural products isolated from plants used in traditional medicine, which have potent anti-plasmodial action *in vitro*, represent potential sources of new anti-malarial drugs.

## Background

The main cause of the worsening malaria situation in recent years has been the spread of drug-resistant parasites. This has led to rising malaria-associated mortality [1]. Anti-malarial drug resistance has emerged as one of the greatest challenges facing malaria control today and has also been implicated in the spread of malaria to new areas and re-emergence of malaria in areas where the disease had been eradicated [2,3]. Drug resistance has also played a significant role in the occurrence and severity of epidemics in some parts of the world. Population movement has introduced resistant parasites to areas previously free of drug resistance. Moreover, in recent years the situation has worsened due to malaria parasites becoming resistant to several anti-malarial drugs [4,5]. This resistance concerns numerous drugs, but is thought to be most serious with chloroquine (CQ), the cheapest and most widely used drug to treat malaria [6].

The use of plants for therapeutic purposes has long been in practice [7]. Medicinal plants have been used in virtually all cultures as a source of medicine [8] and for a long time natural products were the only sources of medication [9]. Several medicinal plants have been used locally to treat malaria infection. Medicinal plants, such as *Momordica charantia* (local name: Ejirin wewe), *Momordica balsamina* (local name: Ejirin), *Ageratum conyzoides* (local name: Imi Eshu), *Diospyros monbuttensis* (local name, Egun Eja), have been used to treat one ailment or the other in Africa, especially Nigeria [10-12].

The urgency generated by drug-resistant strains of malaria parasites has accelerated anti-malarial drug research over the last two decades. While synthetic pharmaceutical agents continue to dominate research, attention has increasingly been directed to natural products [13]. The success of quinine (QN) and artemisinin, isolated from *Artemisia annua* and its derivatives, for the treatment of resistant malaria has focused attention on plants as a source of anti-malarial drugs [14]. Moreover, plants have been the basic source of sophisticated traditional medicine systems for thousands of years and were instrumental in early pharmaceutical drug discovery and industry [15]. The world’s poorest are the worst affected, and many treat themselves with traditional herbal medicines. These are often more available and affordable, and sometimes are perceived as more effective than conventional anti-malarial drugs [16].

Ethnobotanical information about anti-malarial plants used in traditional herbal medicine is essential for further evaluation of the efficacy of plant anti-malarial remedies, and efforts are now being directed towards discovery and development of new, chemically diverse anti-malarial agents [17]. Several rural dwellers depend on traditional herbal medicine for treatment of many infectious diseases [18]. The reputed efficacies of these plants have been experienced and passed on from one generation to the other.

About 75% of the population in Africa does not have direct access to conventional medicine for malaria treatment but they do have access to traditional medicine for treating fevers. Treatment with these remedies has suffered a number of deficiencies; identification of plant extracts may be insecure and the chemical content of extracts may vary considerably [11]. Natural products isolated from plants used in traditional medicine, which have potent anti-plasmodial action *in vitro*, represent potential sources of new anti-malarial drugs [19,20]. It had been advocated that direct crude drug formulation of the herbs following toxicological absolution (after it has been ascertained to be non-toxic) may not only produce dosage forms faster but will also lead to cheaper and more affordable drugs for the communities that need them [21]. This research was carried out in order to increase the database of plants whose extracts can be used in the treatment of malaria.

## Methods

### Study area

**Figure 1:**
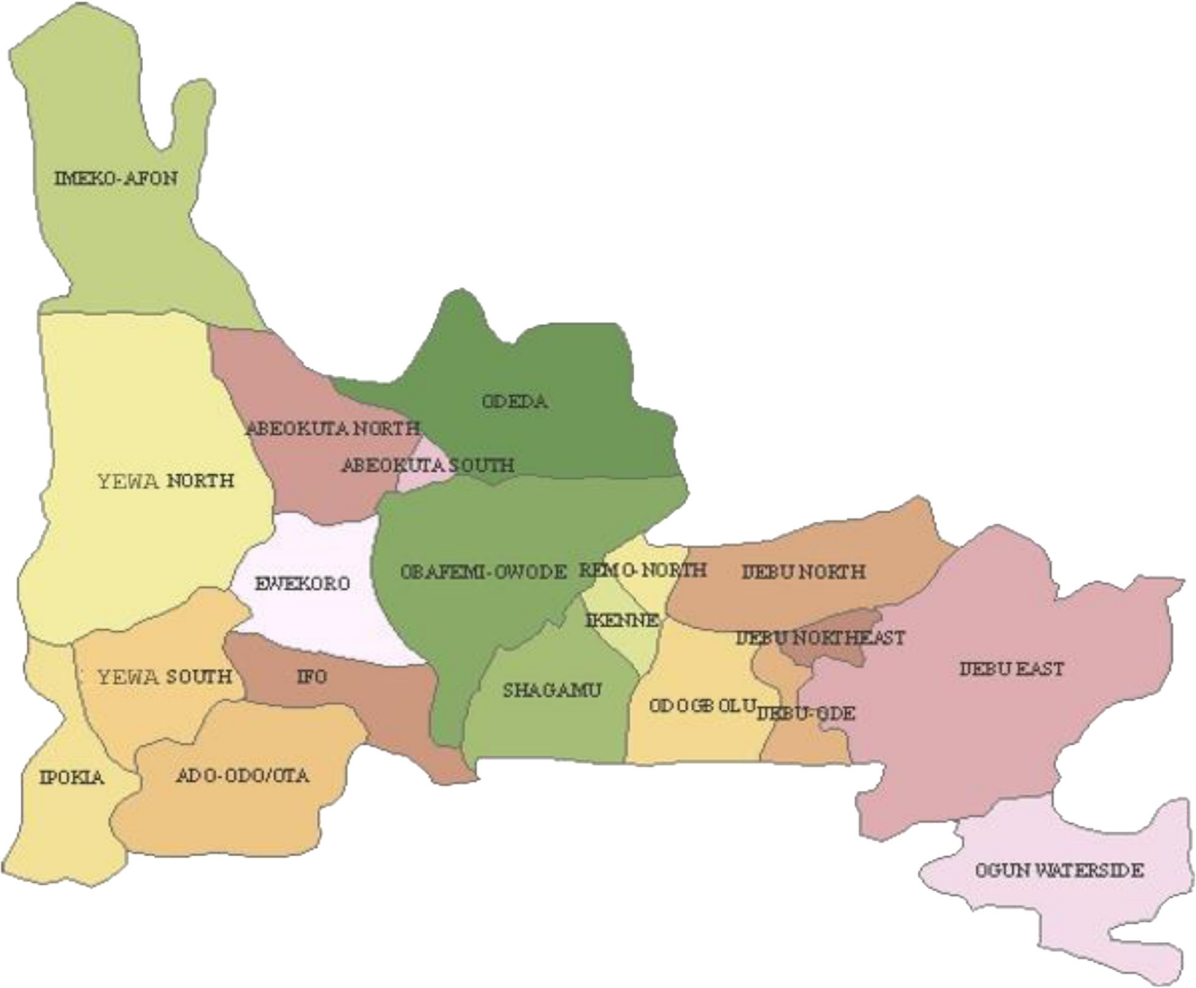
**Map of Ogun State**, **Southwestern Nigeria.**

The study was carried out in Ogun State, south-western Nigeria. The state has a total land mass of 16,409.26 sq km and shares an international boundary with the Republic of Benin to the west and interstate boundaries with Oyo State to the north, Lagos and the Atlantic Ocean to the south and Ondo State to the east (Figure 1). For the purpose of this study, the state was grouped into zones based on the origin of the people, namely, Egba, Ijebu, Yewa/Awori and Remo. One major town from each of these zones (Abeokuta, Ijebu-Ode, Sango-Otta and Sagamu, in that order) was used as a study site. Abeokuta, the capital of Ogun State, lies between latitude 70 10′ N and 70 15′ N and longitudes 30 17′ E and 30 26′ E. Ijebu-Ode lies between 6° 49' N, 3° 56' E. Sango-Otta is situated within the tropical zone, lying between 60° and 47° N of the Equator and 20.33° E and 30.18° E while Sagamu has its geographical coordinates as 6° 51' N, 3° 39' E. Cultured isolates of *Plasmodium falciparum*, totalling 100 in all, were collected from all four towns.

### Sample collection

State general hospitals located in the different zones were used as collection centres. Both in- and outpatients who presented with uncomplicated malaria in the hospitals were recruited for the research work. Blood of the patients was collected using heparin bottles. Subjects were screened according to exclusion/inclusion criteria. Samples used for the study were collected from subjects who had received QN and artemisinin derivatives within the previous seven days, 4-aminoquinolones within the previous 14 days, pyrimethamine or sulphonamides within 28 days or mefloquine within the previous 56 days of blood collection. Blood samples were collected for malaria screening by fingerprick and venipuncture in order to check the presence of asexual parasites in the peripheral smear of patients. Safety procedures were adopted in the collection of fingerprick samples by swabbing the area to be sampled with 70% alcohol and allowing it to dry before collection. The bleeding was done in the hospitals by clinicians and medical laboratory scientists. About 2–5 ml of blood was drawn (venipuncture) using a sterile disposable syringe. The blood samples were collected between April 2008 and June 2009.

### Patients’ characteristics

A total of 4,066 subjects comprising 1,839 males and 2,227 females presenting with malaria in four different zones of Ogun State were recruited into the study. The total number of subjects recruited in Sango-Otta, Abeokuta, Ijebu-Ode and Sagamu were 1,120, 1,116, 995 and 835, respectively. Children between one and 15 years, pregnant women and other adults were included in this study. This is because the majority of malaria cases occur in children under the age of 12 years; pregnant women are also especially vulnerable. The mean age was 19 years, with 93% less than 25 years old.

### Ethical consideration

Scientific and ethical clearance for this work was obtained from the Nigerian Institute of Medical Research – Institutional Review Board (NIMR-IRB) and Covenant University Ethics Committee. The Ogun State Ministry of Health (Hospitals Management Board) was also informed and clearance obtained for this study. Written informed consent was obtained from patients prior to recruitment into this study. Consent for children was provided by parents/guardians while some participants provided the assents.

### Microscopic examination

Giemsa-stained thick and thin films were prepared for the microscopic examination of the malaria parasite. The thin films were fixed with methanol and all films were stained with 3% Giemsa stain of pH 7.0 for 30 min as recommended by WHO [22]. Blood films were examined microscopically using 100X (oil immersion) objectives as described by Cheesbrough [23]. The thick films were used to determine the parasite densities while thin films were used to identify the parasite species and infective stages. Parasite density per microlitre of blood (parasitaemia) was estimated from the thick film, taking the number of leucocytes per microlitre of blood as 8,000.

### Cryopreservation

The infected blood was centrifuged at 959 × g. The supernatant/plasma was removed and cells were suspended in equal volume of cryopreservative. The suspension was distributed into cryotubes and quickly frozen in cryofreezer at −80°C and then transferred into liquid nitrogen (−196°C). The cryopreservative was prepared by adding 28 ml glycerol to 72 ml of 4.2% Sorbitol in normal saline. The solution was sterilized by filtration through a Millipore filter of 0.22 μm porosity.

### Cultivation of parasites

The vial was taken out of the liquid nitrogen tank and thawed quickly in a 37°C water bath. The content was transferred to a centrifuge tube and centrifuged at 959 × g for 10 min. The supernatant was then removed and an equal volume of 3.5% NaCl was added. The suspension was centrifuged again and the supernatant removed. The pellet was washed twice with complete medium supplemented with 15% serum (Albumax). The parasites were cultured using a candle jar in RPMI 1640 medium supplemented with gentamicin solution at 0.01 mg/mL, 25 mM HEPES buffer, 25 mM NaHCO3, and 1% Albumax II maintained in 5% CO_2_ and incubated at 37°C. The estimation of the parasitaemia, as well as parasite visualization before incubation, was done using Giemsa stained blood films on normal light microscopes.

### Preparation of plant extract

Leaves of plants were used. The plants were identified in the Department of Pharmacognosy, Lagos University Teaching Hospital, Lagos. Ten grams of dried extract was dissolved in 50 ml alcohol (95%) 37°C for seven days at room temperature. The alcohol was allowed to evaporate at room temperature. Ten millilitres dimethyl sulphoxide (DMSO) was added to 10 mg of each extract to make 1 mg/ml.

### *In vitro* test

The overall incidence of falciparum malaria as determined by microscopy in the study area was 62.7% (2,250/4,066). Out of the *P. falciparum* samples collected, 100 isolates were successfully cultured and maintained in continuous culture for a minimum of three weeks. Drug samples were prepared in picomole/litre (pM) quantities according to WHO *in vitro* microtest procedure [24,25]. The assay was performed in duplicate in a 96-well microplate, according to WHO method (*in vitro* microtest (Mark III)), which is based on assessing the inhibition of schizont maturation. RPMI 1640 (Gibco BRL, Paisley, UK) was the culture medium used for cultivation of *P. falciparum*[26]. Dilution was prepared from the anti-malarial drugs. 1% DMSO was used to prepare the drug stock. Also a control was set up where only the solvent was present in the well and the parasites were able to undergo schizogony in the control well. Stock solutions of chloroquine sulphate (600 ng/ml) and other anti-malarial drugs (amodiaquine (AQ) = 119 ng/ml, mefloquine (MQ) = 265 ng/ml, quinine (QN) = 462 ng/ml, artesunate (AS) = 360 ng/ml, all prepared according to World Health Organisation’s Standard Operating Procedures were prepared in sterile distilled water (CQ, AQ, MQ and Q or ethanol (sulphadoxine/pyrimethamine (SP) and artemisinin) and used in two-fold dilutions with the culture medium in 96-well culture microplates (Nunc, Denmark) to obtain nine final dilutions (600 to 2.34 ng/ml for CQ) and appropriate dilution factor was determined for the other drugs and the crude plant extracts and the final concentrations prepared by serial dilution were (125, 62.5, 31.25, 15.6, 7.8, 3.9 and 1.95 μg/ml). Negative controls treated by solvent and positive controls (25 nmol/L chloroquine phosphate) were added to each set of experiments for the herbal extracts. Fifty μl which is the final volume per test well, from the parasitized blood mixture media was added to each well in plate and incubated in CO_2_ condition; 5% CO_2_ at 37.5°C for 24–30 hours. After incubation, contents of the wells were harvested and stained for 30 min in a 2% Giemsa solution pH 7.2; after that, the developed schizonts were counted against the total asexual parasite count of 200. The count process was done in duplicate, and the data were analysed by using HN-NonLin V1.1 [27] to determine the IC_50._ The IC_50_ value is defined as the concentration of an anti-malarial drug that inhibits 50% of schizont maturation as compared with the development in drug-free control wells. IC_99_ gives a result that closely approximates a minimum inhibitory concentration (MIC). The MIC is generally defined as the lowest drug concentration that inhibits the development of rings to schizonts.

### Statistical analysis

Data were analysed using Chi-square and ANOVA statistical tests. All the tests were performed at the 95% confidence interval using SPSS version 15.0 software.

## Results

*In vitro* anti-malarial drugs and local herbal extracts sensitivity tests were carried out and the IC_50_ and IC_99_ for each drug were determined. Table 1 shows the mean IC_50_ and IC_99_ values for CQ, AQ, MQ, QN, SP and As. Moreover, the *in vitro* threshold values for anti-malarials have been defined [28]. IC_50_ implies that 50% of the parasite could not mature to schizont stage at that drug concentration while IC_99_ implies that the anti-malarial drugs inhibited 99% of the parasites from maturing to schizont stage at that concentration.

**Table 1:** ***In vitro *** **susceptibility of *Plasmodium falciparum *** **isolates to anti-malarial drugs**

**Drug**	**IC**_**50 **_**mean (nM)**	**IC**_**99 **_**mean (nM)**	**Resistance threshold (nM)**
Chloroquine (CQ)	24.4	164.2	> 160
Amodiaquine (AQ)	6.3	32.4	> 80
Artesunate (AS)	3.2	7.8	> 10.5
Mefloquine (MQ)	42.1	60.8	> 64.0
Sulphadoxine/Pyrimethamine (SP)	55.0	200	> 300
	0.7	2.5	> 4.0
Quinine (Q)	60.3	298.6	> 300

All the selected isolates tested were sensitive to QN, MQ and AS while *in vitro* resistance was observed in CQ, AQ and SP (Table 2). Highest percentage of resistance to CQ (69.8%) was recorded among isolates from Yewa zone while highest percentage of resistance to AQ (30%) was observed in Ijebu zone. Highest percentage of resistance against SP (10%) was recorded in Yewa and Egba zones. A positive correlation was observed between the responses to artemisinin and MQ (P<0.05), artemisinin and Q (P<0.05), QN and MQ (P<0.05) (Table 2). A negative correlation was observed between the responses to CQ and MQ (P>0.05).

**Table 2:** **Zone-wise resistance pattern of *Plasmodium falciparum *** **to anti-malarial drugs**

**Origin**	**Number of isolates cultured**	**CQ res (%)**	**AQ res (%)**	**As res (%)**	**MQ res (%)**	**SP res (%)**	**Q res (%)**
Ijebu	20	9 (45)	6 (30)	0 (0)	0 (0)	1 (5)	0 (0)
Yewa	43	30 (69.8)	4 (9.3)	0 (0)	0 (0)	2 (10)	0 (0)
Egba	25	5 (20)	1 (4)	0 (0)	0 (0)	2 (10)	0 (0)
Remo	12	7 (58.3)	2 (16.7)	0 (0)	0 (0)	0 (0)	0 (0)
**Total**	**100**	**51 (51)**	**13 (13)**	**0 (0)**	**0 (0)**	**5 (5)**	**0 (0)**

CQ **-** chloroquine; AQ **-** amodiaquine; As - artesunate; MQ **-** mefloquine; SP - sulphadoxine/pyrimethamine; Q **-** quinine; res **-** resistance.

The results of *in vitro* anti-malarial activity of the three herbal extracts tested -are as shown in Table 3. Of the selected three herbal extracts, the highest activity was obtained with extract of *D. monbuttensis* (IC_50_ = 3.2 nM) while the lowest was obtained from *M. lucida* (IC_50_ =25nM). *In vitro* resistance against CQ, AQ and SP was observed among Nigerian isolates of *P. falciparum* tested. In the cross-sectional study, an IC_50_ of 24.4 nM and IC_99_ of 164.2 nM was recorded for CQ respectively Moreover, resistance threshold of isolates against sulphadoxine and pyrimethamine was 300 nM and 4.0 nM, respectively. All the isolates tested against MQ, QN and AS in this study were sensitive to the drugs. The sensitivity range observed in the current study was also within the range for sensitivity of isolates to the drug [28]. *In vivo* resistance against CQ, AQ, MQ and SP has been reported by researchers in Nigeria [29,30]. The *in vivo* response of *P. falciparum* to anti-malarial drugs is modulated by a number of factors. These include the pharmacokinetic properties of anti-malarial drugs, innate and acquired immunity in the patient, as well as the complexity of infections in high transmission areas [29]. Several of these factors may contribute to the range of variations in the clinical expression of CQ resistance and *in vitro* resistance patterns.

**Table 3:** ***In vitro *** **susceptibility of *Plasmodium falciparum *** **isolates to local anti-malarial herbs**

**Herbal Drug**	**IC**_**50 **_**nM**
*Momordica charantia* (Ejirin)	12.5
*Morinda lucida* (Oruwo)	25
*Diospyros monbuttensis* (Eegun eja)	3.2

All the isolates tested *in vitro* against artemisinin were sensitive to the artesunate, a derivative of artemisinin. Bioavailability of artemisinin derivatives in vivo amongst other factors has been attributed to treatment failures [31]. AS, which is a derivative of artemisinin, was used for the *in vitro* drug testing in this study because it has been reported that dihydroartemisinin is unstable on drug plates and that artesunate is the most appropriate drug for *in vitro* drug assays due to its stability in predosed plates [32].

High resistance against CQ and AQ were observed among the isolates collected from Yewa and Ijebu zones while SP resistance was not observed in isolates from Remo zone. There was cross-resistance between CQ and AQ as some of the isolates that showed resistance to CQ also showed resistance to AQ in all the zones. Cross-resistance between CQ and AQ has been reported both *in vitro* and *in vivo*. Pradines *et al.*[33] have observed cross-resistance of CQ and AQ in earlier studies. It has also been observed that parasites may quickly develop resistance to AQ in areas where extensive CQ resistance has been documented [34].

There is an increasing acceptance that the ideal approach to anti-malarial treatment is the use of a combination of two or more drugs, rather than a single anti-malarial drug, preferably with an artemisinin derivative as one of the drugs [28,35]. AQ in combination with AS has been introduced as first-line treatment of malaria to replace CQ in Nigeria and other malaria-endemic countries of Africa [36]. Although the role of AS in this combination is to prevent the development of amodiaquine resistance, parasites may quickly develop resistance to AQ in areas where extensive CQ resistance has been documented. In addition, little is known about the mechanism or epidemiology of AQ resistance. Resistant parasites may then likely recrudesce under the selective force of the second drug in the combination and be transmitted to mosquitoes [37]. Therefore, the possibility of increasing selection of AQ-resistant parasites with the increasing use of AQ in combination with AQ in Nigeria cannot be ruled out. Reduced *in vitro* susceptibility is not synonymous with diminished therapeutic effectiveness, but it is the probable first step of an alarming cascade and definitely pleads for increased vigilance and a coordinated and rapid deployment of drug combinations.

Developing countries, where malaria is one of the most prevalent diseases, still rely on traditional medicine as a source for the treatment of this disease. While synthetic pharmaceutical agents continue to dominate research, increasing attention has been directed to natural products [38]. The success of artemisinin, isolated from *Artemisia annua*, and its derivatives for the treatment of resistant malaria has focused attention on plants as a source of anti-malarial drugs [14]. In this study, three crude organic extracts obtained from medicinal plants used in Nigerian folk medicine for the treatment of fever and malaria were tested *in vitro* against *P. falciparum. Diospyros monbuttensis* showed appreciable inhibition to the parasites at all the concentrations used and an IC_50_ of 3.2 nM in the study. *Diospyros monbuttensis,* which is locally used for the treatment of fevers, headaches and stomach disorders [10,11], has not been widely studied. This study represents the first conducted for anti-malarial activity of crude extracts of *D. monbuttensis*. The results confirm that these plants, which are used in traditional medicine against malaria, may possess *in vitro* and significant anti-malarial potential and justify their use in traditional medicine. This observation suggests that the active constituents in the extract may be cytotoxic for *P. falciparum* trophozoites, thereby inhibiting their development to the schizont stage.

An IC_50_ observed for *M. charantia* in this study was 12.5 nM. These observations suggest that the active constituents in the extract might also be cytotoxic for *P. falciparum* trophozoites, thereby inhibiting their development to the schizont stage. The anti-malarial activity of *M. charantia* has been previously reported [38]. They found that the aqueous extract of *M. charantia* leaves showed IC_50_ values less than 100 μg/ml which is in agreement with the observations in this study; the methanolic extract showed moderate activity with IC_50_ = 12.5 nM. *Morinda lucida* also exhibited anti-malarial activity in this study. The IC_50_ of 25 nM observed in this study is comparable with other studies. Also for *M. lucida,* dose-dependent inhibitory outcomes were marked. Awe and Makinde [10], reported the dose-dependent and seasonal variation in the activity of *M. lucida* using both *in vitro* and *in vivo* techniques. *Morinda lucida* was reported to contain anthraquinones, which showed *in vitro* activity against *P. falciparum* and also possess antifungal properties. *Morinda lucida* is used locally in the treatment of yellow fever and jaundice [39].

## Conclusions

*In vivo* studies on these medicinal plants are necessary and should seek to determine toxicity of the active constituents, their side effects, serum-attainable levels, pharmacokinetic properties and diffusion in different body sites. Additional pharmacokinetic investigations are therefore advisable to identify host-related factors, such as poor absorption, accelerated gastrointestinal passage of the test drug, or metabolic peculiarities of some patients, which might lead to a faster-than-normal inactivation or elimination of the test drug.

## Competing interests

The authors declare that there are no competing interests.

## Authors’ contributions

OGI conceived the study and carried out the *in vitro* sensitivity studies; OO participated in the design; AOA participated in the design and the analysis of the *in vitro* sensitivity studies; FEO participated in the design and helped to draft the manuscript; VN participated in the analysis of the *in vitro* sensitivity studies; ELO participated in the design and analysis of the *in vitro* sensitivity studies; AAA participated in the collection and extraction of plants; AOI participated in the collection of plants and statistical analysis. All authors read and approved the final manuscript.
